# Explore the Therapeutic Composition and Mechanism of Schisandra chinensis-Acorus tatarinowii Schott on Alzheimer's Disease by Using an Integrated Approach on Chemical Profile, Network Pharmacology, and UPLC-QTOF/MS-Based Metabolomics Analysis

**DOI:** 10.1155/2022/6362617

**Published:** 2022-07-11

**Authors:** Jian Chen, WenQian Hao, Chengqin Zhang, MeiRong Cui, Yue Sun, Ying Zhang, Jing Wang, TingTing Mou, Shuo Gu, TianTian Zhao, Binbin Wei

**Affiliations:** Central Laboratory, School of Pharmacy, China Medical University, No. 77 Puhe Road, Shenyang 110122, China

## Abstract

**Background:**

Alzheimer's disease places a heavy economic burden to healthcare systems around the world. However, the effective treatments are still lacking. Traditional Chinese medicines (TCM) of Schisandra chinensis and Acorus tatarinowii Schott have the pharmacological effects of sedation and neuroprotection and have been clinically proven to be effective in the treatment of AD. However, their main anti-Alzheimer's compounds and functional mechanisms remain unclear.

**Purpose:**

To elucidate the main therapeutic components and possible mechanisms of Sc-At in AD using a comprehensive strategy combining metabolomics and network pharmacology.

**Methods:**

First, the UPLC-QTOF/MS method was used to identify the main chemical constituents of Schisandra chinensis and Acorus tatarinowii Schott alcohol extracts in *vitro* and *in vivo*. Secondly, the theoretical active ingredients, targets, and pathways of Sc-At for AD treatment were predicted by network pharmacology methods. Finally, plasma metabolomics were detected by UPLC-QTOF/MS to analyze the differential metabolites and metabolic pathways related to Sc-At. Based on the analyses above, the anti-AD mechanism of Sc-At was explored.

**Results:**

A total of 95 chemical components were identified in Sc-At extracts in *vitro*, and 34 prototype drug components were detected in rat plasma; network pharmacology screening identified 14 drug components in line with the principle of Lipinski, of which 10 were present for *in vitro* drug composition analysis. For these 10 components, 58 AD disease targets were predicted, and 85 AD-related KEGG signaling pathways were enriched. Six core biomarkers of Sc-At (*cis*-8,11,14,17-eicosatetraenoic acid, prostaglandin H2, sphingosine 1-phosphate, enol-phenylpyruvate, 3-methoxytyrosine, and pristanoyl-CoA) were regulated to a normal state during the treatment of AD.

**Conclusion:**

The mechanism of Sc-At for the treatment of AD can be achieved by the effect of the 10 compounds of Sc-At on TNF, MAPK8, MAPK14, PTGS1, and other targets, thereby affecting arachidonic acid metabolism, neurotransmitters, and sphingolipid metabolism.

## 1. Introduction

Alzheimer's disease (AD) is a neurodegenerative disease whose pathogenesis has yet to be elucidated and for which there is no complete cure. New drug development for Alzheimer's disease remains critical. “Drug pair” usually consists of two herbs, which are paired with their different properties in the clinical prescriptions of TCM. Schisandra chinensis-Acorus tatarinowii Schott is one of the commonly used drugs in the treatment of dementia with kidney deficiency and phlegm. “Kidney deficiency and phlegm” are the pathogenesis of dementia in Chinese traditional medicine based on years of clinical experience combined with TCM theory; TCM has developed a theory of an independent cognitive impairment system; the pathogenesis of this impairment includes deficiency, phlegm, and stasis evidence, involving a range of organs, including the brain, kidneys, heart, liver, and spleen [1]. Guided by this mechanism, the TCM approach to treating dementia is to use herbs that tonify the kidneys and remove phlegm and phlegm, so that the body can achieve a relative balance [2]. Schisandra chinensis belongs to Magnoliaceae plants, and the crude extracts and isolated pure lignin components of its fruits have been reported to protect damaged neurons and enhance cognitive function [3]. The dried rhizome of Acorus tatarinowii Schott is a kind of phlegm-resolving and orifice-opening herb. Studies have found that its effective components can improve cognitive dysfunction caused by Alzheimer's disease [3]. The combination of Schisandra chinensis and Acorus tatarinowii Schott can tonify the kidney and resolve phlegm, thus having definite curative effect on dementia of kidney deficiency and phlegm stasis type [4]. We searched several databases related to Alzheimer's disease. According to the frequency of use and meta-analysis results, we found that Schisandra chinensis and Acorus tatarinowii Schott are the key drugs in TCM prescriptions for AD treatment. However, there are few studies on the combination of these two herbs in treating AD and the effective components and mechanism of Sc-At in AD treatment have not been clarified, posing obstacles to the TCM development.

In recent years, network pharmacology and system biology share many concepts with the holistic philosophy of TCM, which facilitates the systematic research to overcome disease with complex mechanisms such as cancer [5]. Network pharmacology can integrate massive data, conduct virtual screening according to TCM prescriptions and corresponding symptoms, and reveal the molecular correlation between compounds and complex diseases at multiple scales [6]. Metabonomics research is based on the overall metabolic profiling of biological samples (such as urine, plasma, or tissue), and it emphasizes the holistic study of the multiparameter metabolic responses of biological systems to external stimuli (including pathological stimuli and drug therapy). This research can be used to monitor the dynamic changes of endogenous small molecule metabolites in organisms [7, 8].

**Figure 1:**
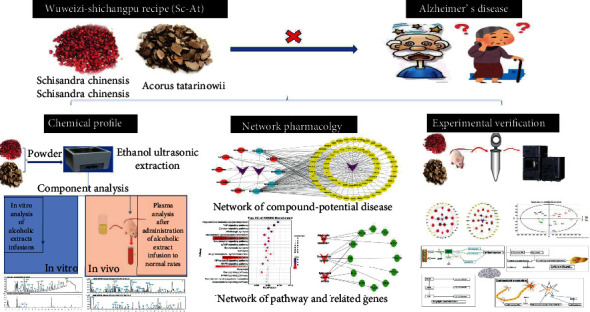
Study design of the present study.

In this study, the UPLC-QTOF/MS nontarget metabolomics method was used to study the potential biomarkers of rat AD model plasma before and after Sc-At treatment. Besides, the interaction network between the theoretically active components of Sc-At and the target of Alzheimer's disease was constructed by network pharmacology. Then, these active components were analyzed *in vitro* and verified *in vivo* by UPLC-QTOF/MS. In short, the mechanism of Sc-At in treating Alzheimer's disease was studied by combining network pharmacology and metabolomics, laying the foundation for the modernization of TCM. The design of this study is shown in Figure 1.

## 2. Materials and Methods

### 2.1. Chemicals and Reagents

Sc-At was purchased from Shenyang Tianyitang (Liaoning, China) and identified by Professor Chen Zaixing (Pharmacy, Chinese Medical University). Schisandra is the dried ripe fruit of Schisandra chinensis (Turcz.) Baill. of the Magnoliaceae family, which is distributed in northeastern China. In traditional Chinese medicine, it has the effect of astringent and astringent, benefiting Qi, nourishing the kidney, and nourishing the heart. Acorus tatarinowii Schott (Latin name:Acorus tatarinowii)belongs to the family Araceae, Acorus calamus grass-like perennial herbs, medicinal parts for the rhizome, and is distributed in Asia, including northeast India, northern Thailand, China, south of the Yellow River, and other regions. The voucher samples were stored in the author's laboratory. UPLC-MS mass spectrometry grade methanol and acetonitrile were purchased from Merck (Darmstadt, Germany). The ultrapure water was supplied by Watson's (Guangzhou, China). Mass spectrometry grade formic acid was purchased from Thermo Fisher Scientific (China) Co., Ltd. Leucine enkephalin was purchased from Sigma-Aldrich (St. Louis, MO, USA). Analytical grade aluminum chloride was purchased from Tianjin Guangfu Fine Chemical Research Institute, and D-galactose was provided by Hefei Biotechnology Company. Rat A*β* amyloid protein 25–35 was purchased from Beijing Biosynthesis Biotechnology Co., Ltd. Schisandrol A and Schizandrin A were purchased from Shanghai Yuanye Biotechnology Co., Ltd; *α*-Asarone and Beta-Asarone were purchased from Chengdu Zhibiaohua Pure Biotechnology Co., Ltd; Schizandrin B was purchased from Sichuan Vicki Biotechnology Co., Ltd. Reference compounds were higher than 98% as determined by UPLC. TNF-*α* kit was purchased from Beijing Chenglin Biotechnology Co., Ltd. Other reagents and chemicals were analytical.

### 2.2. Schisandra chinensis and Acorus calamus Herb Identification

Liquid chromatographic runs were performed on an ACQUITY UPLC system (Waters Corporation, Milford, USA). Chromatographic separation was achieved with an ACQUITY UPLCTM T3 column (100 mm × 2.1 mm, 1.8 *μ*m particles) (Waters Corporation, Milford, USA) at 30°C. Mobile phase A was acetonitrile, and mobile phase B was water. A mobile phase was achieved with gradient elution using a complex gradient, as the follows: 5% A ⟶ 55% A at 0–1.5 min, 55% A ⟶ 80% A at 1.5–3 min, 80% A ⟶ 80% A at 3–8 min, 80% A ⟶ 55% A at 8–10 min, and 55% A ⟶ 5% A at 10–10.5 min. Symmetrical and efficient peaks were obtained at a flow rate of 0.4 ml/min with a sample injection volume of 10 *μ*l, PDA (210 nm-400 nm) scan. Standard stock solutions of the 5 reference standards (Schisandrol A, Schizandrin A, *α*-Asarone, Beta-Asarone, and Schizandrin B) were prepared by dissolving them in methanol. They were then diluted to 0.2 mg/ml; the stock and diluted solutions were stored at -4°C.

### 2.3. Schisandra chinensis-Acorus tatarinowii Schott Sample Preparation

Three alcohol extraction samples of the Schisandra chinensis, Acorus tatarinowii, and Sc-At (1 : 1) were prepared by ultrasonic alcohol extraction method. The Sc-At were crushed by a 40-mesh sieve. The powder obtained was accurately weighed 50 g each and then mixed with 10 times the volume of 90% ethanol. The mixture was soaked for 1 h and then ultrasonically extracted for 1 h. The filtrate was vacuum filtered, and the residue was added into 10 times 90% ethanol and extracted twice. Then, the filtrate was combined, concentrated, and freeze-dried. An appropriate amount of freeze-dried Sc-At powder was added into distilled water to prepare a solution with a concentration of 1.5 g/ml, and then, the mixture was put into an ultrasonic disperser at 4°C to prepare a soluble solution for further use. Besides, the freeze-dried powder was dissolved in methanol and diluted to an appropriate multiple. The diluted solution was centrifuged at 12000 rpm at 4°C for 10 minutes and filtered through a 0.22 *μ*m membrane.

### 2.4. Schisandra chinensis and Acorus calamus Analytical Conditions

The chromatographic conditions were as follows: column temperature 40°C, flow rate 0.4 ml/min, and injection volume 3 *μ*l. The ACQUITY UPLCTM T3 column (Waters Corporation, Milford, USA) was applied for chromatographic separation. The mobile phase consisted of water (solvent A) and acetonitrile (solvent B) containing 0.1% formic acid solution. Schisandra chinensis elution gradient is as follows: 0-1 min 10% B; 1-3 min 10-45% B; 3-8 min, 45-60% B; 8-18 min, 60-90% B; 18-20 min, 10% B; 20-23 min, 10% B; Acorus tatarinowii Schott positive mode elution gradient: 0-1 min 5% B; 1-28 min, 5-95% B; 28-31 min, 5% B; 31-32 min, 5% B; negative mode elution gradient: 0-1 min 5% B; 1-13 min, 5-40% B; 13-18 min, 40-75% B; 18-20 min, 5% B, 20-25 min, 5% B.

### 2.5. Establishment of Chemical Constituent Library

The chemical constituents of Sc-At were obtained from TCMSP (http://tcmspw.com/tcmsp.php), and the internal libraries of compounds covering all Sc-At herbs were established in a Microsoft Excel table with relevant literature. The exact mol files were collected by Chemical book and ChemSpider corresponding to CAS number and then imported into UNIFI (US waters company version 1.7) software to create a database. Then, peak extraction and automatic matching identification of compounds were performed and compared with the chromatographic and mass spectrometry information of reference (blank solvent/blank plasma) to confirm the structure and corresponding fragment information of the matched compounds. The filter function of UNIFI mass spectrometry analysis software “Unknown unique” was deduced as a potential candidate compound according to the standard of ppm less than 10. Only compounds that were compared with standard or characteristic fragment ions were finally selected as Sc-At chemical components.

### 2.6. Network Pharmacology Analysis

The compound information of Schisandra chinensis and Acorus calamus is retrieved from the Pharmacological Database Analysis Platform of Traditional Chinese Medicine System (http://tcmspw.com/tcmsp.php). According to the results of *in vivo* and *in vitro* component analysis of Sc-At, the candidate compounds were screened combined with the pharmacokinetic parameters of oral bioavailability (OB) ≥ 30% and drug similarity (DL) ≥ 0.18. Further target prediction was carried out according to the candidate compounds. The UniProt database (http://www.uniprot.org/) was used to match the predicted targets. The DAVID biological information database (http://david.abcc.ncifcrf.gov/) was used to predict related pathways. The GeneCards database (https://www.genecards.org/) was used to predict AD disease targets. The results of network pharmacology and subsequent experimental results were compared and integrated to explore the mechanism of Sc-At in AD treatment.

### 2.7. Animal Treatment and Sample Collection

Eight-month-old male Wistar rats (weight 200 g ± 20 g) were provided by Liaoning Changsheng Biotechnology Co., Ltd (China Liaoning). Room temperature was 24 ± 2°C, and humidity was 55 ± 5%. A 12-hour light/dark cycle was set to give the animals free access to standard diet and water. The research was approved by the Animal Care and Use Committee of China Medical University (Shenyang, China) (Experimental Ethics No. CMU2019257). After a week of environmental adaptation, they were randomly divided into four groups, with 6 rats in each group. The first three groups were the blank group (B group), the model group (M group), and the Schisandra-Corus calamus group (Sc-At group), respectively. The dosage was calculated according to the practice guide for dose conversion between rats and human. The previous study has determined that the M group and Sc-At group were given D-galactose 120 mg/kg/d and aluminum chloride 40 mg/kg/d, once daily for four weeks. From the second week, the Sc-At group began to accept the Sc-At treatment at a dosage of 2.5 g/kg/d, and normal administration (modeling drug) was continued in the M group until twelve weeks. Group B was fed normally. The M group and Sc-At group were given a bilateral intracerebroventricular injection of A*β*25-35 10 *μ*g/side. On the 99th day of model replication, the Morris water maze was used to evaluate the learning and memory ability of all groups. Six days after the behavioral examination, rats in each group were decapitated, and blood and tissue samples were collected on the 106th day (see Figure 1 for specific experimental steps).

### 2.8. Preparation of Plasma Samples

The plasma samples stored at -80°C were taken out; 100 *μ*l of each sample was absorbed; 300 *μ*l of precooled (-20°C) methanol was added, vortex shaken for 1 min, left at -20°C for 20 min, and centrifuged at 4°C for 20 min (14000 rpm). The supernatant was taken, dried with nitrogen, and stored at -80°C. Each portion of the dried sample was dissolved in 120 *μ*l complex solution (methanol : water = 1 : 1) and centrifuged at 4°C for 10 min (12000 rpm).

### 2.9. UPLC-QTOF/MS Analysis

Xevo G2-XS QTof-MS (Waters, Milford, USA) was adopted to analyze the plasma samples together with the Water ACQUITY UHPLC system controlled by MassLynx (version 4.1, Walter, Milford, USA). Firstly, 3 *μ*l of sample solution was injected into an ACQUITY UPLCTM T3 column (Walsch, Milford, USA) at 40°C with a flow rate of 0.4 ml/min. The mobile phase consisted of water (solvent A) containing 0.1% formic acid solution and acetonitrile (solvent B). Each sample was eluted at a flow rate of 0.4 ml/min for 23 minutes. Elution gradient design is as follows: 0-2 min, 95% B; 2-21 min, 95%-5% B; 21-23 minutes, 95% B. The optimum conditions for mass spectrometry analysis were as follows: source temperature of 120°C and desolventizing gas temperature of 500°C. The capillary voltage was 3.0 kV, and the sampling cone voltage was 45.0 V, respectively. To obtain accurate masses, a locked spray interface at a flow rate of 10 *μ*l/min and a locked mass of leucine enkephalin at a concentration of 200 pg/*μ*l were used to monitor the positive/negative ion pattern ([M+H]^+^ 556.2771/([M-H]^−^ 554.2615).

### 2.10. Data Processing and Analysis

UPLC-QTOF/MS raw data (MassLynx (version 4.1, Walter, Milford, USA) acquired) of plasma samples were imported into metabolomics software (Progenesis Qi (US waters company version 2.3)). The MS data such as peak extraction, peak matching, and peak alignment were preprocessed in Progenesis Qi (Waters) 2.3. After the data were normalized by total ionic strength, the list of interesting features including retention time, *m*/*z* value, and normalized peak intensity were used for principal component analysis (PCA) and orthogonal least squares discriminant analysis (OPLS-DA) and further confirmed by ANOVA. Then, *t*-tests were performed on the peak intensities of different metabolites using statistical software to compare and confirm the changes of biomarkers between different experimental groups. *P* < 0.05 was set as the critical value. The quality control (QC) samples of all samples were taken as a small sample group and injected at the beginning, periodic interval, and the end of the sample queue under the same chromatographic condition. The quality data were collected to evaluate the sensitivity and stability of the instrument performance based on the quality accuracy, retention time stability, and variation coefficient. Finally, the data matrices, including retention time, *m*/*z*, and peak intensity, were obtained. The identified metabolites were analyzed in metabolic websites (11 databases including Kegg, WikiPathways, Reactome, and HumanCyc) and MetaboAnalyst attached to Qi.

## 3. Results

### 3.1. UPLC Identification Results of Schisandra chinensis and Acorus calamus

**Figure 2:**
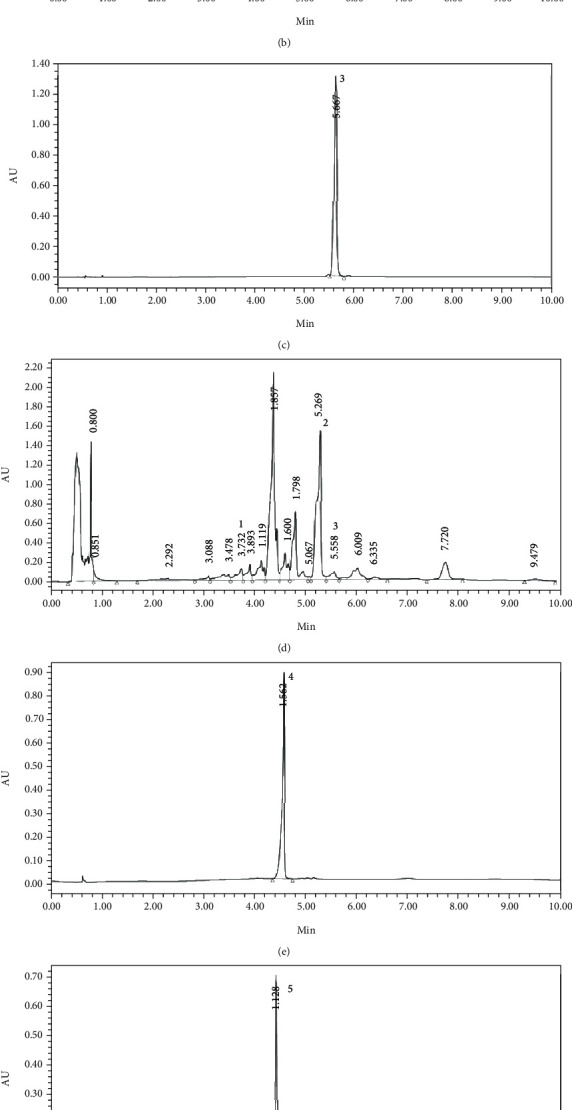
Typical chromatograms for the determination of five bioactive compounds in alcoholic extracts of Schisandra chinensis and Acorus sinensis: (a) Schisandrol A, (b) Schizandrin A, (c) Schizandrin B, (d) Schisandra chinensis, (e) *α*-Asarone, (f) Beta-Asarone, and (g) Acorus tatarinowii Schott; peak 1: Schisandrol A; 2: Schizandrin A; 3: Schizandrin B; 4: *α*-Asarone; 5: Beta-Asarone, respectively.

Figure 2 shows standard chromatograms A (Schisandrol A), B (Schizandrin A), C (Schizandrin B) corresponding to the LC analysis chromatogram D of the Sc sample and standard chromatograms E (*α*-Asarone) and F (Beta-Asarone) corresponding to the LC analysis chromatogram of the At sample.

### 3.2. Determination of Chemical Constituents of Sc-At by UPLC-QTOF/MS

**Figure 3:**
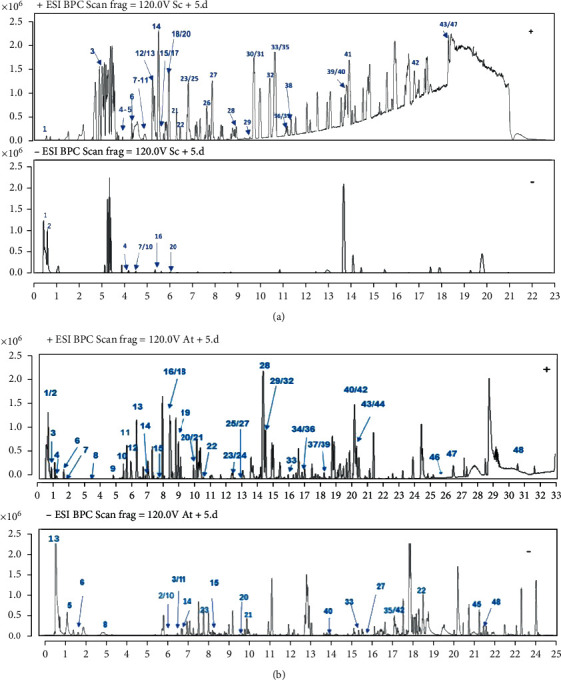
Representative base peak chromatograms of active components of Schisandra chinensis and Acorus tatarinowii: (a) Schisandra chinensis; (b) Acorus tatarinowii.

**Figure 4:**
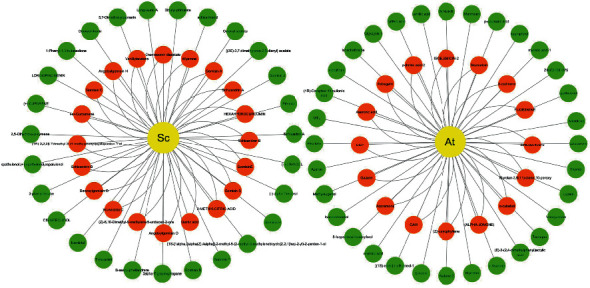
The results of in vitro and in vivo component analysis of Schisandra chinensis and Acorus tatarinowii Schott alcohol extracts. The yellow circle represents the traditional Chinese medicine, the red circle represents the ingredients screened in vivo and in vitro, and the green represents the ingredients screened only in vitro.

In this study, the alcohol extract samples of Schisandra chinensis and Acorus tatarinowii Schott were tested with UPLC-QTOF/MS technology that was used to optimize the chromatographic and mass spectrometric conditions. According to the self-built database of UNFI, the drug components were identified as follows: Schisandra chinensis has a total of 47 compounds, including 17 lignans, 12 volatile oils, 10 fatty acids, 4 alkaloids, and 4 miscellaneous compounds. The Acorus tatarinowii Schott contains a total of 48 compounds, including 9 terpenoids, 6 flavonoids, 4 phenylpropanoids, 2 lignins, 6 fatty acids, and 21 heterocyclic compounds. Among these compounds, 20 prototypes in Schisandra chinensis and 16 in Acorus tatarinowii Schott were identified in the plasma of normal rats after administration. The determination of compounds is based on the accuracy of excimer ions such as [M-H]^−^, [M+HCOO]^−^, [M+CH_3_COO]^−^, [M+H]^+^, and [M+Na]^+^, and the ions recorded in the internal library (Figure 3).  Figure 4 provides the results of *in vitro* and *in vivo* component analyses of two Chinese medicines. Tables S1–4 provide tR (min), identification, negative ions (*m*/*z*), positive ions (*m*/*z*), and plant sources.

### 3.3. Network Pharmacology Analysis

**Figure 5:**
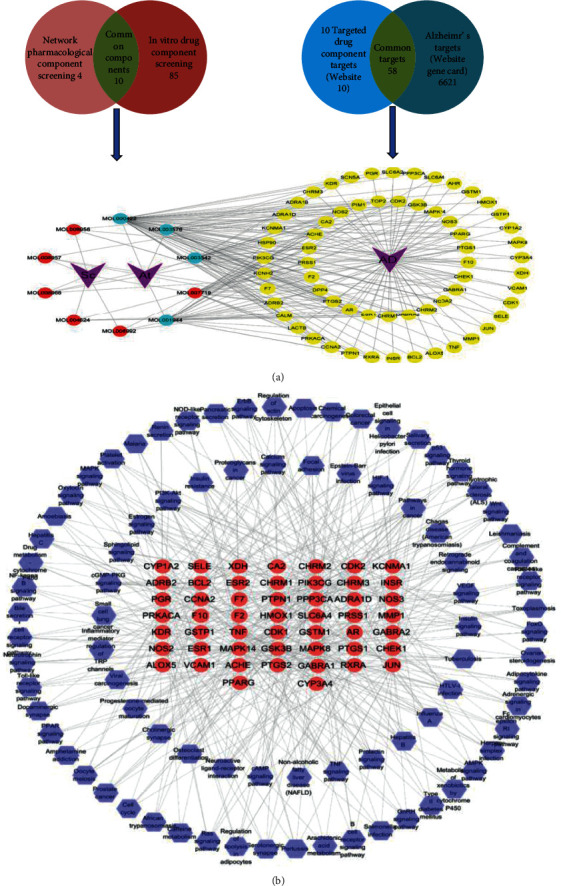
Summary of network pharmacology. (a) The signaling pathways of the 10 drug components and their corresponding targets. (b) Targets and their corresponding pathway.

Based on two criteria, i.e., oral bioavailability ≥ 30% and drug similarity ≥ 0.18, 14 active compounds in Sc-At were retrieved from databases and related literature. Combined with the results of *in vitro* component analysis of Sc-At, the disease targets of 10 compounds shared by the two were predicted (Tables 1 and 2), and 67 Sc-At prediction targets were obtained. By matching them with Alzheimer's disease-related targets obtained through GeneCards, we finally identified 58 Sc-At potential targets for AD treatment. Then, the potential drug component-target-disease-pathway network constructed with Cytoscape 3.7.2 is shown in Figure 5.

### 3.4. Metabolic Data Analysis of Plasma Samples

**Figure 6:**
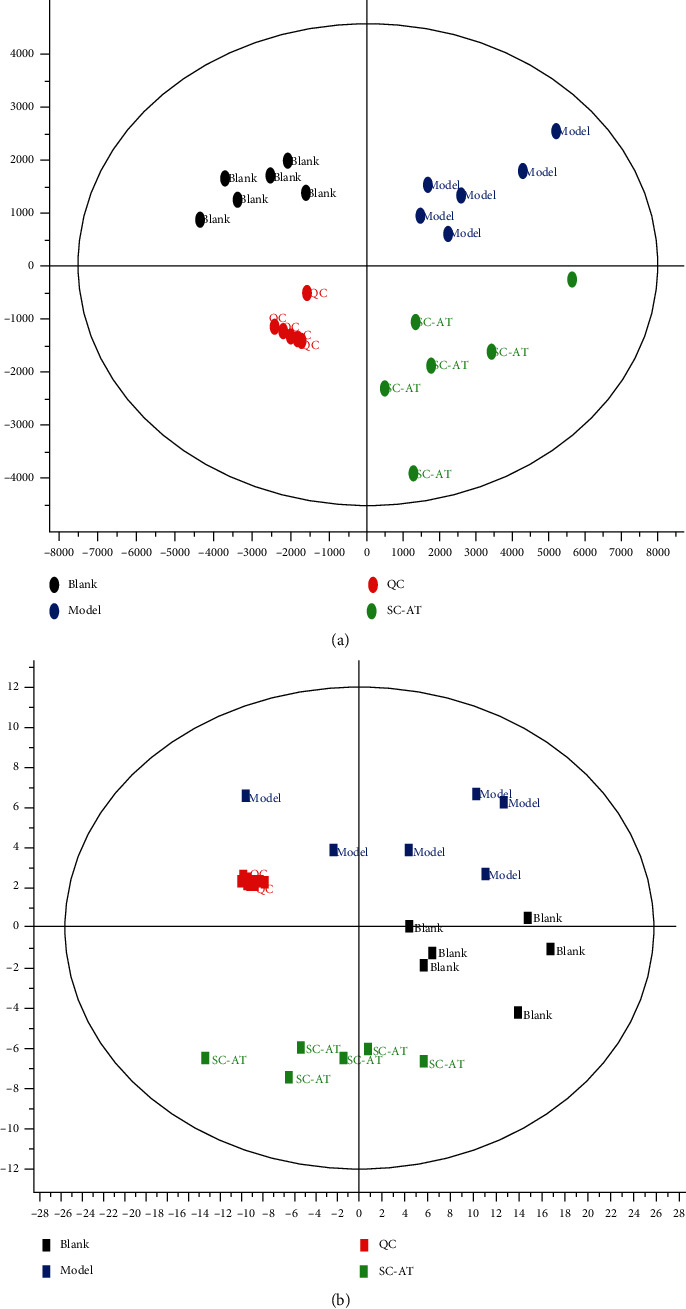
Principal component analysis score plots derived from rat plasma metabolic profile in positive mode (a) and negative mode (b). Notes: *n* = 6, per group.

The metabolomics data of plasma samples were obtained by UPLC-QTOF/MS. The mass spectrum information of the blank group, model group, Sc-At group, and QC group was imported into Progenesis QI v2.3 (nonlinear dynamics, Newcastle, UK) for multiple statistical analysis. After all the mass spectrometry peak information of each sample was preprocessed with mass spectrometry data such as peak extraction, the obtained list of compounds containing retention time, *m*/*z* values, and normalized peak intensity values were subjected to principal component analysis (PCA) and orthogonal least squares discriminant analysis (OPLS-DA) in EZinfo software. The points of each color in the PCA figure represent a sample. As shown in Figure 6, each group of samples can be separated, especially QC (quality control) samples. These results showed that the serum endogenous metabolites of the three groups were significantly different, and the LC-MS detection system was stable during the experiment. This method can intuitively describe the differences in metabolic patterns and clustering results among different groups.

The OPLS-DA model established in this study was cross-validated on Qi software to detect the predictive ability of the model for the experiment. The results were M vs. B (R2Y = 0.9964, Q2 = (0.9017). The parameters in this study showed that the model was reliable and stable, which could be used to find metabolic markers in the next step.

**Figure 7:**
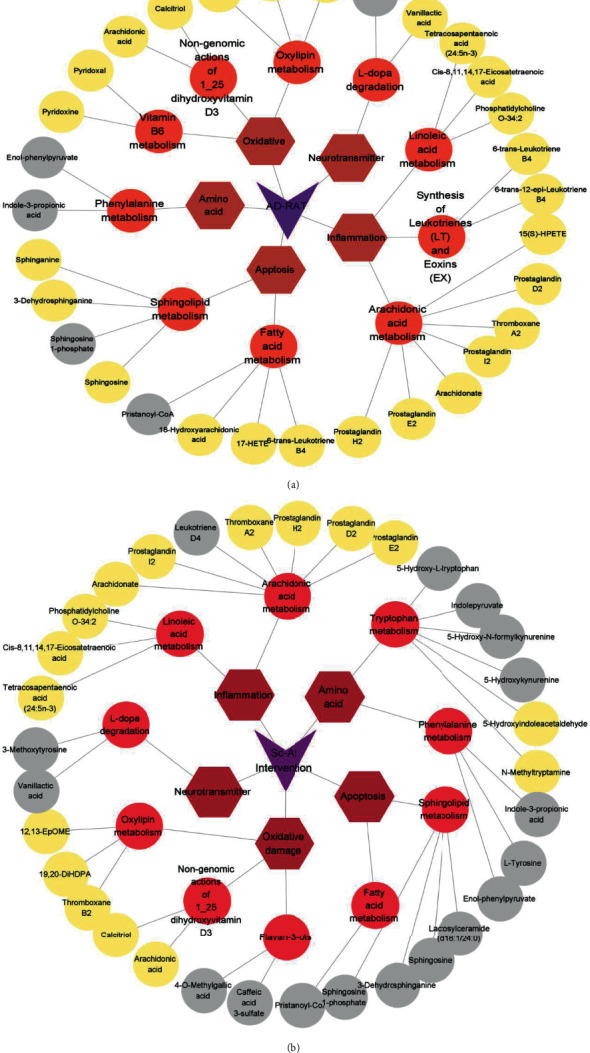
Summary of metabolite classification in metabolomics. (a) Blank vs. model (AD rat) differential metabolites. (b) Sc-At vs. model differential metabolites. The grey circle represents the metabolites with reduced content, the yellow circle represents the metabolites with increased content, the orange circle represents the pathway, the brown hexagon represents the category to which they belong, and the purple triangle represents the subgroup to which they belong.

Plasma nontargeted metabolomics preliminarily screened 10 metabolic pathways in the rat AD model, involving 43 related metabolites. 30 (25 up/5 down) and 33 (17 up/16 down) differential endogenous metabolites were identified between the model and blank and model and Sc-At groups, respectively. These metabolites mainly involve sphingolipid metabolism, unsaturated fatty acid biosynthesis, arachidonic acid metabolism, and vitamin B6 metabolism (Figure 7). They are mainly involved in inflammation, oxidative stress, amino acid metabolism, and neurotransmitter degradation metabolism. We comprehensively analyzed these metabolites and concluded that 12 metabolites might be the key biomarkers for Sc-At in the treatment of AD (Table 3). Recent evidence has suggested that Alzheimer's disease also includes a large number of neuronal loss, inflammation, extensive DNA damage, considerable mitochondrial dysfunction, impaired energy metabolism, and chronic oxidative stress, which are consistent with the findings of this study that the associated metabolic dysfunction is a possible cause and marker of Alzheimer's disease [9].

### 3.5. Metabolite Pathway Correlation Analysis

**Figure 8:**
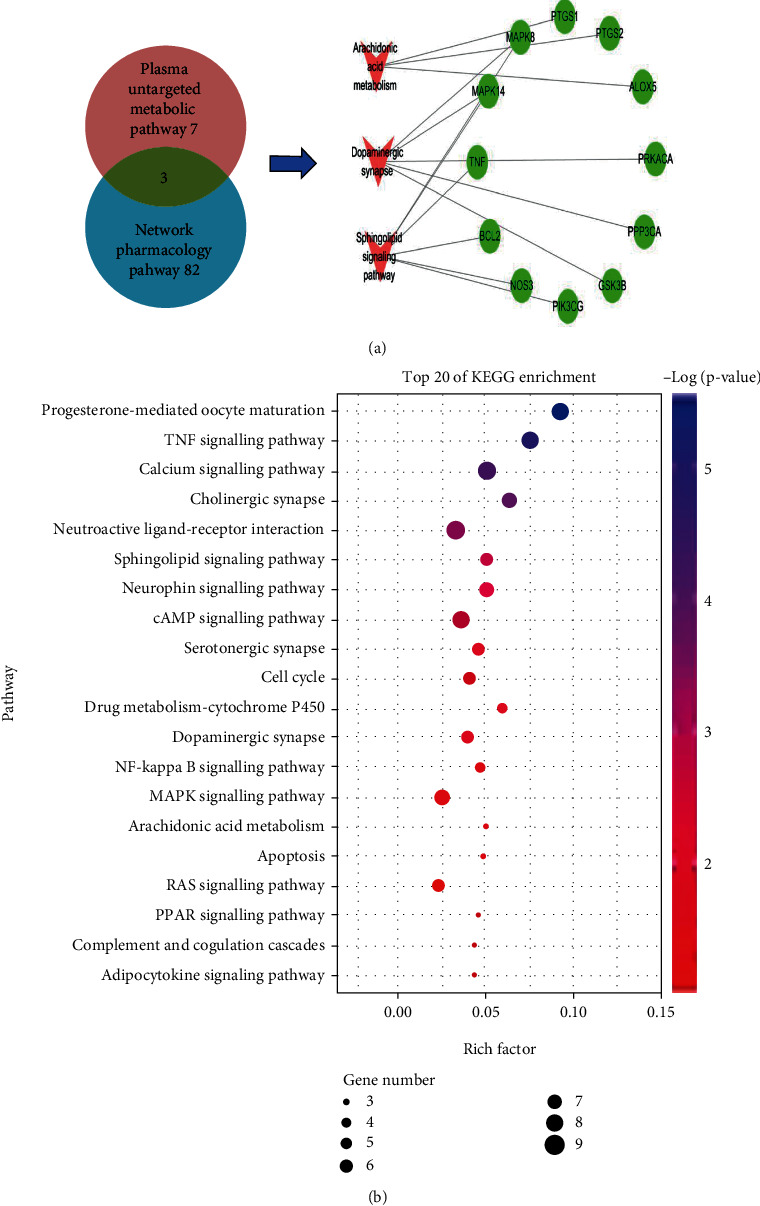
Network pharmacology and metabolomics result association analysis presentation. (a) Network pharmacology and metabolomics common pathways and targets. (b) Location of the common pathway in the network pharmacology KEGG pathway.

In this study, the potential targets of 10 compounds in Sc-At for the treatment of Alzheimer's s disease were analyzed by network pharmacology. The results showed that 85 KEGG signaling pathways were enriched, which mainly included the sheath lipid signaling pathway (hsa04071), arachidonic acid (hsa05200), TNF signaling pathway (hsa04668), and cholinergic synapse (hsa04725). The enrichment pathways of differential metabolites in the nontarget metabolic group mainly include sphingolipid metabolism, dopamine degradation, and arachidonic acid metabolism. The three mutually confirmed pathways were the sphingolipid signaling pathway (sphingolipid metabolism), arachidonic acid metabolism, and cholinergic synapse (L-dopa degradation). These metabolic pathways are mainly related to biological processes such as inflammation, neurotransmitters, and apoptosis (Figure 8).

**Figure 9:**
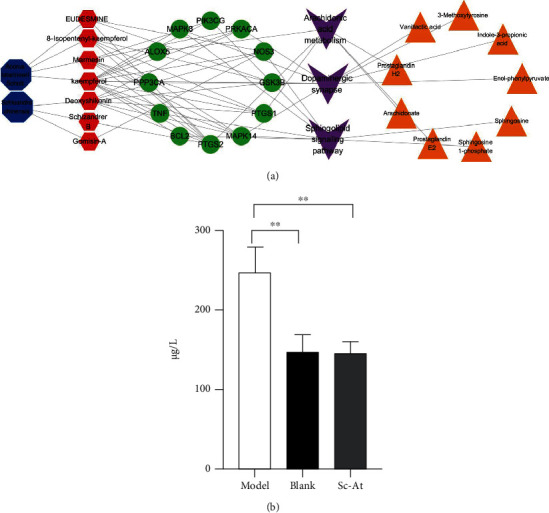
(a) Network pharmacology-metabolomics association analysis component-target-pathway-metabolite schematic diagram. Notes: the large and small octagon denotes medicinal herbs (SC, AT) and corresponding ingredients, red denotes target points, purple square denotes the metabolic pathway, and yellow triangles represent metabolites. (b) Expression of TNF-*α* in rat plasma (*n* = 3, ^∗^*P* < 0.05).

This study concluded that SC-AT treatment of AD may be achieved by affecting 9 key metabolites through 7 main components, acting on 12 related targets, and affecting 9 key metabolites through three pathways (Figure 9(a)).

The results of our network pharmacology analysis showed that in the theoretical signaling pathway of Sc-At treatment of AD, kaempferol, a component in calamus, acts on the sphingolipid signaling pathway through tumor necrosis factor, and this factor has significant statistical significance (Figure 9(b)).

## 4. Discussion

The prevailing hypotheses regarding the pathogenesis of AD include A*β* protein deposition, Tau protein hyperphosphorylation, cholinergic neuronal damage, oxidative stress, and neuroinflammation. Studies have reported [10] that Schisandra chinensis, Acorus calamus, and their active ingredients may prevent neurological disorders.

**Figure 10:**
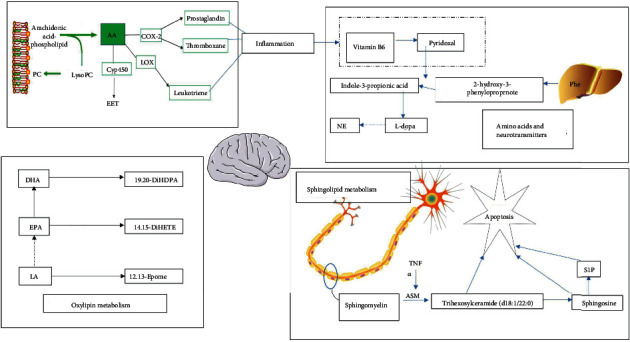
Metabolic pathway diagram. Notes: green represents enzymes associated with the pathway, red represents decreased levels in the administered group compared to the model, and blue represents increased levels.

The sphingolipid signaling pathway, dopaminergic synapse, and arachidonic acid metabolic pathway are the pathways predicted by network pharmacology and validated in plasma untargeted metabolomics of the rat AD model in this study. Inflammatory mediators are chemical factors that participate in and mediate the inflammatory response in Alzheimer's disease. Studies have found that elevated serum arachidonic acid levels increase the risk of AD [11, 12]. In this study, compared with the blank group, the content of PGH2 in the model group was lower than that in the blank group. After treatment with St-Ac, the content of PGH2 in the administration group was higher than that in the model group, indicating that St-Ac may affect the level of PGH2 by affecting the content of arachidonic acid in the blood, so it has a certain therapeutic effect on AD. The specific metabolic process is shown in Figure 10 (metabolic pathway diagram). Also, some studies have pointed out that the markers of chronic inflammation are negatively correlated with the plasma PLP (pyridoxamine) level, and plasma PLP participates in multiple enzyme reactions to affect the metabolism of sphingolipids or neurotransmitters [13]. The same result has been obtained by this study. Compared with the blank group, the production of inflammatory factors such as arachidonic acid in the model group is increased, resulting in the decrease of vitamin B6 level and the increase of dopamine production. Our study found a negative correlation between sphingolipid metabolite S1P and PLP levels, which may be the potential mechanism of Sc-At in the treatment of Alzheimer's disease.

Ceramide, ceramide-1-phosphate (C1P), and 1-phosphate sphingosine 1-phosphate (S1P) play a variety of roles in the development of the nervous system, which is a critical biological activity, and the acquisition of mature neuron phenotypes. Under the influence of sphingomyelinase, sphingomyelin is degraded to ceramide on the cell membrane; then, ceramide is converted to sphingosine by ceramide enzyme, and finally, sphingosine is phosphorylated to S1P [14]. S1P precursors (sphingosine and ceramide) have proapoptotic effects on damaged neurons. The prosurvival activity of S1P in the brain highlights its potential as a therapeutic target for neurodegenerative diseases [15, 16]. In this study, compared with the blank group, the level of S1P decreased and the level of sphingosine increased in the model group, and the levels of S1P and sphingosine decreased after administration. The results showed that the sphingolipid metabolic balance of AD model rats was broken, and SC-AT improved the symptoms of AD by regulating the specific metabolic balance of S1P and sphingosine.

Amino acids play an important role in many metabolic pathways. Amino acid neurotransmitters, a kind of important neurotransmitters in the brain, regulate the excitation or inhibition of central neurons and are closely related to neuronal information transmission, nutritional development, cognitive activities, learning, and memory. Phenylalanine (Phe) is a type of *α*-amino acid, which is an essential amino acid for the human body. The intake of phenylalanine in the liver is catalyzed by phenylalanine hydroxylase to generate tyrosine. Tyrosine in the adrenal medulla and nerve tissue can be catalyzed by tyrosine hydroxylase to generate levodopa, which is then decarboxylated to generate dopamine, hydrolyzed to generate norepinephrine, and methylated to generate epinephrine, which becomes a neurotransmitter or hormone. Studies have shown that a decrease in neurotransmitters such as dopamine in the brain tissue may be one of the main causes of neurological diseases. In this study, the Sc-At administrative group significantly reduced the degradation of L-dopa and increased the content of dopamine in the brain, which may be part of the drug's approach to treating AD.

Studies have found that the dopamine system is closely linked to cognitive function in patients with AD and can predict the rapid development of AD [17]. It has been proved that drugs targeting the dopamine system can change the aggregation state of A*β* protein, and the peptide oxidation of A*β* aggregation changes depends on the oxidation degree of dopamine [18]. Among the abnormal neurotransmitters studied in AD, the dopaminergic system has been intensively studied as a key neurotransmitter system related to mood and cognition, and the therapeutic effects of some dopaminergic system drugs have been validated in AD, suggesting that the dopaminergic active system may be a reasonable target for pharmacological intervention in AD [19]. Numerous studies [20] have shown that compounds such as Schisantherin A, Angeloylgomisin O, and Schisantherin A can improve learning and memory impairment in AD mice by participating in the modulation of neurotransmitters (e.g., dopamine) and their metabolite changes in the brain. The dopamine biosynthetic signaling pathway is coregulated by multiple compounds in Sc-At. In addition to the 10 prototypical drug components screened, other components such as Schisantherin A prevented 6-OHDA-induced DA neuron loss and restored motor behavior deficits in zebrafish. In this study, compared with the model group, the Sc-At administration group significantly reduced the levels of 3-methoxytyrosine and vanillactic acid, thereby increasing the content of dopamine in the brain by reducing the degradation of dopamine precursor levodopa, which may be one of the ways to treat AD.

The results of several experiments [21] showed that estrogen can inhibit neuroinflammation and antioxidant and improve memory impairment, and the lignan contained in Sc-At extract belongs to a kind of phytoestrogen, which has the effects of scavenging free radicals in the body, antioxidant, and binding estrogen receptor to interfere with cancer-promoting effects and has a positive effect on AD prevention and treatment; this is similar to the results of our study.

Tumor necrosis factor (TNF), also known as TNF*α*, can directly kill tumor cells and has no significant cytotoxic effect on normal cells [22]; however, a key molecule in inflammation that is a proinflammatory cytokine is TNF-*α*. Early reports have shown that TNF-*α* levels are elevated in the cerebrospinal fluid of AD patients compared to cognitive normal controls, and high levels of TNF-*α* activate the NF-*κ*B inflammatory signaling pathways, further exacerbating the pathological features of A*β* deposition and tau hyperphosphorylation. Increasing evidence suggests that dysregulation of cytosolic calcium (Ca^2+^) signaling regulating neuronal homeostasis may induce synaptic defects and promote A*β* plaques and neurofibrillary tangles, playing an important role in the pathogenesis of AD [23].

The cholinergic system [24] has an important role in the study of Alzheimer's disease in the improvement of functions such as memory, learning, and cognition. Cholinergic synapse and ligand-receptor interaction are the main pathways affecting neurotransmitter transmission in the cholinergic nervous system. Therefore, we hypothesize that the mechanism of Sc-At treatment of AD may be achieved by regulating neuronal damage in the cholinergic nervous system of AD through the above two signaling pathways.

Although several authoritative hypotheses have been identified that may play a role in the pathogenesis of AD and other neurodegenerative pathologies, the therapeutic effects of drugs developed to target a single hypothesis have been suboptimal; a systems biology- (histology, systems pharmacology) based approach to characterize the complexity of AD pathophysiology is important for the development of drugs for the treatment of AD. In this study, the mechanism of Sc-At for AD treatment was investigated by network pharmacology and metabolism techniques, and it was concluded that Sc-At for AD treatment may be achieved by affecting inflammation, antioxidant, neurotransmitter metabolism, and neuronal damage.

## 5. Conclusion

In this study, network pharmacology and metabolomics strategies were used to evaluate the effective active components of Sc-At and the theoretical active targets and metabolic pathways of the anti-AD effect. A total of 10 theoretical active anti-AD effects were identified from the ethanol extracts of Schisandra chinensis and Acorus gramineus. A total of 12 potential biomarkers of Sc-At for the treatment of AD were identified. Combined with the analysis results of network pharmacology, it was found that sphingolipid metabolism, dopamine metabolism, and arachidonic acid metabolism may be the key metabolic pathways of Sc-At for the treatment of Alzheimer's disease. TNF, MAPK8, MAPK14, PTGS1, and PTGS2 may be the targets of Sc-At. This study provides a reliable basis for further exploring the mechanism of Sc-At for the treatment of Alzheimer's disease.

## Figures and Tables

**Table 1:** 10 drug ingredient information in network pharmacology.

Mol ID	Molecule name	Molecule formula	OB (%)	DL	Herbs
MOL004624	Longikaurin A	C_20_H_28_O_5_	47.72	0.53	Schisandra chinensis
MOL007719	Deoxyshikonin	C_21_H_22_O_6_	73.85	0.18	Schisandra chinensis
MOL008956	Angeloylgomisin O	C_28_H_34_O_8_	31.97	0.85	Schisandra chinensis
MOL008957	Schizandrer B	C_28_H_34_O_9_	30.71	0.83	Schisandra chinensis
MOL008968	Gomisin-A	C_23_H_28_O_7_	30.69	0.78	Schisandra chinensis
MOL008992	Wuweizisu C	C_22_H_24_O_6_	46.27	0.84	Schisandra chinensis
MOL001944	Marmesin	C_14_H_14_O_4_	50.28	0.18	Acorus tatarinowii Schott
MOL003542	8-Isopentenyl-kaempferol	C_20_H_18_O_6_	38.04	0.39	Acorus tatarinowii Schott
MOL003576	EUDESMINE	C_22_H_26_O_6_	52.35	0.62	Acorus tatarinowii Schott
MOL000422	Kaempferol	C_15_H_10_O_6_	41.88	0.24	Acorus tatarinowii Schott

**Table 2:** 10 drug ingredient information.

Peak	tR (min)	Identification	Formula	Positive ion				Negative ion			
				Quasimolecular mass (Da)	Observed mass (Da)	Calculated molecular ion	ppm	Quasimolecular mass (Da)	Observed mass (Da)	Calculated molecular ion	ppm
1	9.86	Longikaurin A	C_20_H_28_O_5_	[M+Na]^+^	348.1936	348.2903	0	—	—	—	—
2	10.8	Deoxyshikonin	C_16_H_16_O_4_	[M+H]^+^	273.1119	273.1127	-0.9	—	—	—	—
3	4.06	Angeloylgomisin O	C_28_H_34_O_8_	[M+H]^+^	499.2316	499.2332	—	[M+HCOO]^−^	543.2235	543.223	-0.1
4	8	Schizandrer B	C_28_H_34_O_9_	[M+NH^4^]^+^	515.2274	532.2547	—	—	—	—	—
5	6.16	Gomisin-A	C_23_H_28_O_7_	[M+Na]^+^	439.1726	439.1733	—	—	—	—	—
6	9.1	Wuweizisu C	C_22_H_24_O_6_	[M+H]^+^	385.1646	385.1651	0.1	—	—	—	—
7	5.22	Marmesin	C_14_H_14_O_4_	[M+NH_4_]^+^	264.1218	264.1236	-4.8	—	—	—	—
8	9.66	8-Isopentenyl-kaempferol	C_20_H_18_O_6_	—	—	—	—	[M-H]^−^	353.1043	333.1279	3.4
9	8.47	EUDESMINE	C_22_H_26_O_6_	[M+H]^+^	387.18	387.1808	-0.5	—	—	—	—
10	0.6	Kaempferol	C_15_H_10_O_6_	[M+NH_4_]^+^	304.0804	304.0821	-3.8	—	—	—	—

**Table 3:** Identification and change trends of biomarkers.

HMDB	Measured mass	Formula	Mass error (ppm)	Compounds	Pathway	Change fold	
						SG/MG	MG/BG
HMDB0002177	305.2482	C_20_H_32_O_2_	2.14	*ci*s-8,11,14,17-Eicosatetraenoic acid	Linoleic acid metabolism	↑^∗∗^	↓^∗∗^
HMDB0006323	359.2949	C_24_H_38_O_2_	1.36	Tetracosapentaenoic acid (24:5n-3)	Linoleic acid metabolism	—	↑^∗∗^
HMDB0001381	375.2138	C_20_H_32_O_5_	-1.1	Prostaglandin H2	Arachidonic acid metabolism	↑^∗∗^	↓^∗∗∗^
HMDB0000277	379.2491	C_18_H_38_NO_5_P	0.99	Sphingosine 1-phosphate	Sphingolipid metabolism	↓^∗∗^	↑^∗∗∗^
HMDB0000252	300.2891	C_18_H_37_NO_2_	-1.94	Sphingosine	Sphingolipid metabolism	↓	—
HMDB0012225	164.0481	C_9_H_8_O_3_	4.77	Enol-phenylpyruvate	Phenylalanine metabolism	↓^∗∗^	↑^∗∗∗^
HMDB0002302	207.1131	C_11_H_11_NO_2_	1.35	Indole-3-propionic acid	Phenylalanine metabolism	↓^∗∗^	↑^∗∗^
HMDB0001903	417.3379	C_27_H_44_O_3_	3.74	Calcitriol	Vitamin B6 metabolism	—	↓^∗∗^
HMDB0004702	297.2436	C_18_H_32_O_3_	4.1	12,13-EpOME	Oxylipin metabolism	—	↓^∗∗∗^
HMDB0001434	212.0927	C_10_H_13_NO_4_	4.59	3-Methoxytyrosine	L-dopa degradation	↓^∗∗∗^	↑^∗∗∗^
HMDB0000913	230.1031	C_10_H_12_O_5_	3.64	Vanillactic acid	L-dopa degradation	↓^∗∗^	↓^∗∗^
HMDB0002057	1079.401	C_40_H_70_N_7_O_18_P_3_S	-3.42	Pristanoyl-CoA	Fatty acid metabolism	↓^∗∗^	↑^∗∗∗^
HMDB0002177	305.2482	C_20_H_32_O_2_	2.14	*cis*-8,11,14,17-Eicosatetraenoic acid	Linoleic acid metabolism	↑^∗∗^	↓^∗∗^

Notes: *n* = 6, per group; data are expressed as mean ± SEM. The up (↑) and down (↓) arrows represent the relative increasing or decreasing trend of the metabolites;“—” represents no significant differences between SG and MG; a change trend of MG vs. NG, for a *t*-test, ^∗∗∗^*P* < 0.001 and ^∗∗^*P* < 0.05 (SG: Sc-At group; MG: model group; BG: blank group).

## Data Availability

The data used to support the findings of this study are available from the corresponding author upon request.

## Abbreviations

AD: Alzheimer's disease
DL: Drug likeness
ESI: Electrospray ionization
MAPK: Mitogen-activated protein kinase
OB: Oral bioavailability
OPLS-DA: Orthogonal least squares discriminant analysis
PCA: Principal component analysis
PGH2: Prostaglandin
H2; PTGS: Posttranscriptional gene silencing
Sc-At: Schisandra chinensis-Acorus tatarinowii Schott
TCM: Traditional Chinese medicine
TNF: Tumor necrosis factor
UPLC-QTOF/MS: Ultraperformance liquid chromatography quadrupole-time of flight/mass spectrometer
VIP: Variable importance in projection.

## Supplementary Materials

Supplementary Materials Table S1: the in vitro component identification of Schisandra chinensis alcohol extract. Table S2: the identification of blood components in vivo after intragastric administration of Schisandra chinensis alcohol extract to rats and orbital blood collection. Table S3: the in vitro component identification of Acorus tatarinowii Schott alcohol extract. Table S4: the identification of blood components in vivo after intragastric administration of Acorus tatarinowii Schott alcohol extract to rats and orbital blood collection. Figure S1: specific experimental steps.
